# Challenges Posed by Embryonic and Anatomical Factors in Systematic Lymphadenectomy for Endometrial Cancer

**DOI:** 10.3390/jcm9124107

**Published:** 2020-12-19

**Authors:** Damaris Freytag, Julian Pape, Juhi Dhanawat, Veronika Günther, Nicolai Maass, Georgios Gitas, Antonio Simone Laganà, Leila Allahqoli, Ivo Meinhold-Heerlein, Gaby N. Moawad, Matthias Biebl, Liselotte Mettler, Ibrahim Alkatout

**Affiliations:** 1 Department of Obstetrics and Gynecology, University Hospital of Schleswig-Holstein, Campus Kiel, Arnold-Heller-Strasse 3, 24105 Kiel, Germany; Damaris.Freytag@uksh.de (D.F.); JulianMaria.Pape@uksh.de (J.P.); juhidhanawat@gmail.com (J.D.); Veronika.Guenther@uksh.de (V.G.); Nicolai.Maass@uksh.de (N.M.); profmettler@gmx.de (L.M.); 2 Department of Obstetrics and Gynecology, University Hospital of Schleswig-Holstein, Campus Lübeck, Ratzeburger Allee 160, 23538 Lübeck, Germany; g.gitas@gmail.com; 3 Department of Obstetrics and Gynecology, Center of Excellence in Minimally-Invasive Gynecology (COEMIG), “Filippo Del Ponte” Hospital, University of Insubria, Piazza Biroldi 1, 21100 Varese, Italy; antoniosimone.lagana@uninsubria.it; 4 Endometriosis Research Center, Iran University of Medical Sciences (IUMS), Tehran 1593747811, Iran; lallahqoli@gmail.com; 5 Department of Obstetrics and Gynecology, University of Giessen, Klinikstraße 33, 35392 Gießen, Germany; ivo.meinhold-heerlein@gyn.med.uni-giessen.de; 6 Department of Obstetrics and Gynecology, Division of Minimally-Invasive Gynecologic Surgery, George Washington University Hospital, Washington, DC 20037, USA; gnmoawad@gmail.com; 7 Department of Surgery, Campus Charité Mitte and Campus Virchow-Klinikum, Charité–Universitätsmedizin Berlin, 10117 Berlin, Germany; matthias.biebl@charite.de

**Keywords:** endometrial cancer, lymphadenectomy, embryology, sentinel lymph node mapping, indocyanine green, PMMR, technical aspects

## Abstract

Lymph node involvement has been shown to be one of the most relevant prognostic factors in a variety of malignancies; this is also true of endometrial cancer. The determination of the lymph node status is crucial in order to establish the tumor stage, and to consider adjuvant treatment. A wide range of surgical staging practices are currently used for the treatment of endometrial cancer. The necessity and extent of lymph node dissection is an ongoing controversial issue in gynecological oncology. Lymph node surgery in endometrial cancer is technically challenging, and can be time consuming because of the topographic complexity of lymphatic drainage as such, and the fact that the lymph nodes are directly adjacent to both blood vessels and nerves. Therefore, profound and exact knowledge of the anatomy is essential. Sentinel lymph node mapping was recently introduced in surgical staging with the aim of reducing morbidity, whilst also obtaining useful prognostic information from a patient’s lymph node status. The present review summarizes the current evidence on the role of lymph node surgery in endometrial cancer, focusing on the embryological, anatomical, and technical aspects.

## 1. Introduction

Endometrial cancer is the most common gynecological cancer in developed countries; more than 380,000 new cases are reported each year worldwide [1]. In accordance with the growing age of the population and the increasing prevalence of metabolic syndromes and obesity, data from the U.S. suggest a consistent increase in the prevalence of this disease [2]. As the symptoms usually occur quite early, the majority of patients (71%) present with early-stage malignancies [3]. The overall survival (OS) rates are high for stage I of the disease: more than 90% of patients are free of disease at five years after surgery [3,4]. The mainstay of treatment for this cancer is surgery [5,6,7,8]. Total hysterectomy with or without bilateral salpingo-oophorectomy and lymphadenectomy permits the removal of the cancer, as well as its classification on the basis of its histological subtype, grading, myometrial invasion, and lymph node status. Traditionally, surgery is performed via open laparotomy. Since the introduction of laparoscopy in the 1990s, a number of studies have shown that laparoscopic treatment is a safe and feasible option for the management of endometrial cancer. Laparoscopy is associated with a lower rate of postoperative complications than open laparotomy [9]. The largest randomized trial comparing laparoscopy with laparotomy was the LAP2study in 2009, which consisted of patients with clinical stage I-IIA uterine cancer who underwent hysterectomy, salpingo-oophorectomy, pelvic and para-aortic lymphadenectomy, and pelvic cytology. The LAP2 study showed that laparoscopic surgical staging is safe and feasible in terms of short-term outcomes, and is associated with shorter hospital stays and fewer complications [10]. The long-term results of this trial were published in 2012 [11]. In the laparoscopy group, the authors observed a small increase in cancer recurrence. However, the overall survival was identical in both groups [11].

According to the ESMO–ESGO–ESTRO guidelines, minimally-invasive surgery is recommended for the surgical management of low- (stage I endometrioid, grade 1–2, <50% myometrial invasion, no lymphovascular space invasion) and intermediate-risk endometrial cancer (stage I endometrioid, grade 1–2, ≥50% myometrial invasion, no lymphovascular space invasion) [12].

Histologically, endometrioid adenocarcinomas are the most common type of endometrial cancer. Other subtypes, including adenosquamous, clear-cell, and serous carcinomas are associated with a poorer prognosis. They are typically more aggressive, and in a more advanced FIGO stage. Endometrial cancer grows into the surrounding tissue, most frequently the myometrium and the cervix. Lymphatic spread also occurs, mainly to the pelvic lymph nodes and then to the para–aortic nodes. The probability of lymph node metastasis across all of the FIGO stages is 15% [12].

Lymph node involvement has been shown to be one of the most relevant prognostic factors in a variety of malignancies. The determination of the lymph node status is crucial in order to establish the tumor stage, and to consider adjuvant treatment [13]. In two randomized trials published in 2008 and 2009, patients who received systematic pelvic lymphadenectomy were compared to those who did not undergo node dissection; the studies demonstrated no benefit in terms of recurrence-free survival (RFS) or overall survival (OS). Since this time, the necessity of lymph node dissection is an ongoing controversial issue in gynecological oncology [14,15,16,17,18]. The extent of para-aortic lymphadenectomy is also a debated issue. Sentinel lymph node mapping is gaining increasing importance in recent times.

The introduction of minimally-invasive surgery in routine gynecology and oncology has minimized the invasive nature of many operations in the female pelvis and retroperitoneum. As mentioned earlier, minimally-invasive surgery benefits patients in many ways. Robotically-assisted surgery is the most dynamic advancement of minimally-invasive surgery, and a significant step in terms of technical evolution. The better visualization of the field of surgery by means of 3D technology and the extension of surgical instruments to 7 degrees of freedom permit the use of minimally-invasive surgery even in complex situations such as obesity or severe adhesions.

A wide range of surgical staging practices are currently used for the treatment of endometrial cancer. The spectrum of lymph node surgery includes sentinel lymph node mapping and systematic pelvic or pelvic and para-aortic lymph node dissection. Peritoneal mesometrial resection (PMMR, initiated by M. Höckel) involves therapeutic pelvic and para-aortic lymphadenectomy (tLNE).

The aim of the present review is to summarize the current evidence on the role of lymph node surgery in endometrial cancer, focusing on the anatomical, embryological, surgical, and technical aspects. The technical challenge of MIS (conventional laparoscopy and robotic surgery) is compared with conventional laparotomy.

## 2. Anatomy

### 2.1. Lymphatic Drainage and Blood Supply to the Uterus and Uterine Adnexa

**Figure 1:**
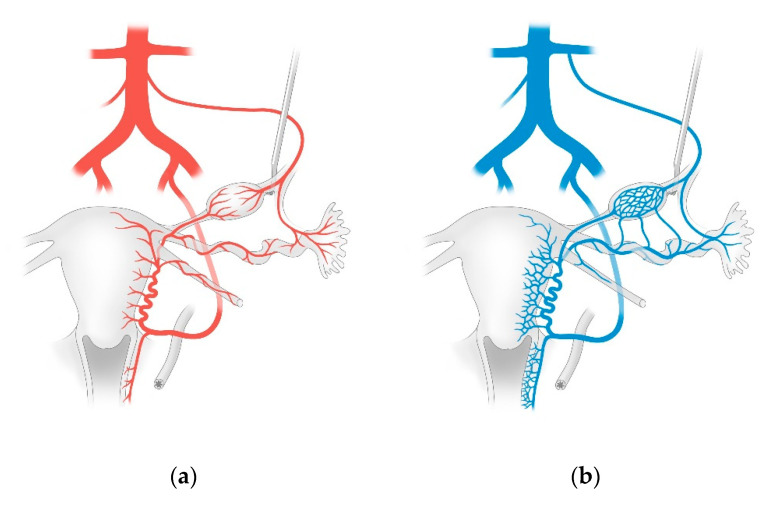
Blood supply: the (**a**) arterial and (**b**) venous blood supply to the female genital organs. The figure schematically shows that the ureter crosses underneath the uterine artery 1–2 cm lateral to the cervix and lateral vaginal fornix. In the majority of cases, only one single uterine artery exists on each side. Additionally, multiple uterine veins of different sizes drain the uterine venous plexus. These veins frequently do not directly follow the course of the uterine artery, but often pass underneath the ureter [19].

**Figure 2:**
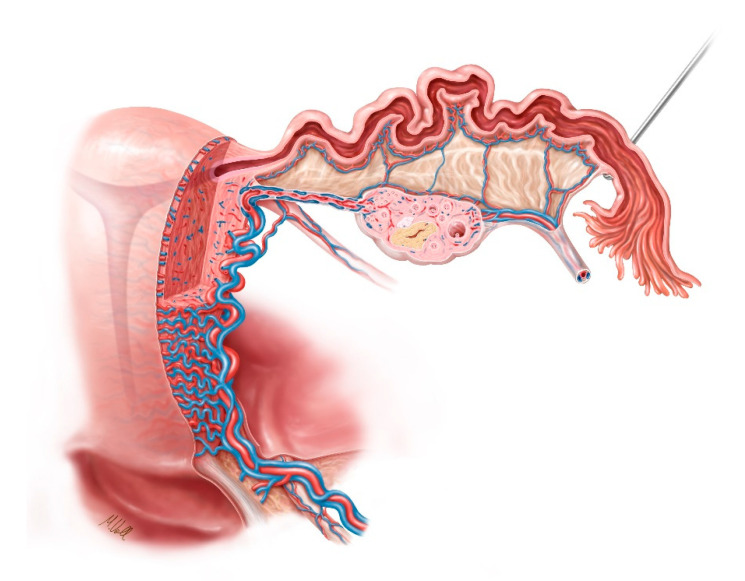
Schematic illustration of the blood flow in the inner female genital organs.

**Figure 3:**
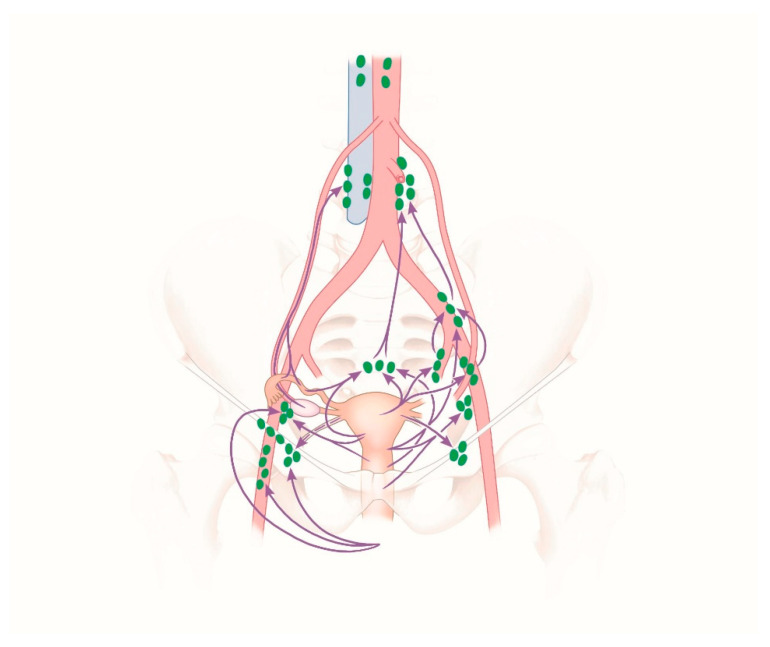
Lymphatic drainage and regional lymph nodes of the female genital organs. The individual lymph node groups are marked green; violet arrows indicate the lymphatic flow from the respective anatomical region.

Lymphatic drainage follows the blood supply of the respective organs. As the arterial supply to the uterus and uterine appendages is derived from two different sources, lymphatic drainage is provided by the pelvic as well as the para-aortic pathways. Due to the peculiar ontogenetic anatomy of the female genital tract, the lymphatic drainage is multidirectional and complex, and has a direct impact on the surgical strategy for endometrial cancer [19]. Lymph node dissection in this area can be time consuming because of the topographic complexity of lymphatic drainage as such, and the fact that the lymph nodes are directly adjacent to both blood vessels and nerves [19]. As such, profound and exact knowledge of the anatomy of the corresponding areas is essential. The following section is focused on anatomy (this is summarized in schematic form in Figure 1, Figure 2 and Figure 3).

### 2.2. Blood Supply

As shown in Figure 1a, the arterial blood to the ovaries is provided by the respective uterine and ovarian arteries. The ovarian artery arises from the aorta, below the renal artery, and courses in the suspensory ligament of the ovary in order to reach the ovarian hilum. Before entering the medulla, it divides into a tubal branch. In the mesosalpinx, this branch forms an anastomosis with the eponymous branch of the uterine artery. The uterine artery arises from the internal iliac artery and courses in the connective tissue from the lateral pelvic wall to the lateral part of the cervix. It crosses the ureter before entering the bladder and then branches off in a T-shaped manner. In the caudal aspect, it forms a vaginal branch lying in the paracolpos, and in the cranial aspect, it forms the markedly convoluted helicine branch which goes on to the mesometrium. Concentric ‘rings’ of blood vessels, or so-called arcuate branches, arise from both branches and supply the uterus and vagina [19,20].

### 2.3. Venous Blood Flow

As shown in Figure 1b, the ovarian vein arises at the ovarian hilum from a markedly-tortuous venous plexus known as the ovarian venous plexus. This drains on the right side into the inferior vena cava. On the left side, the ovarian vein flows into the left renal vein. The venous blood flow from the uterus and the vagina starts at the pairwise uterovaginal plexus, which lies in the lateral aspect of the uterus and vagina in the parametrium and the paracolpos. This plexus drains into the internal iliac vein through the uterine vein [19,20]. Figure 2 illustrates the blood supply to the female genital organs.

### 2.4. Lymphatic Drainage

The lymphatic drainage in the lower part of the vagina and the external genital organs is achieved through the superficial inguinal lymph nodes flowing into the external iliac lymph nodes. The lymphatic drainage from the upper part of the vagina is achieved mainly through the internal iliac lymph nodes. From the uterine cervix, lymphatic pathways reach the lymph nodes in the region of the large pelvic vessels and their branches: the external and internal iliac lymph nodes, the obturator lymph nodes, and the sacral lymph nodes. The subsequent lymph node chain, namely the common iliac lymph nodes and the lumbar lymph nodes, is located around the common iliac artery and the abdominal aorta. Lymphatic pathways from the uterine fundus, the fallopian tubes, and the ovaries course in the suspensory ligament of the ovary (also known as the infundibulopelvic ligament) along the ovarian vessels to the lumbar lymph nodes. The lymphatic pathways from the tube and the uterine corpus extend from the round ligament of the uterus through the deep inguinal ring to the lymph nodes in the groin, namely the superficial inguinal lymph nodes, and further on to the external iliac lymph nodes [19,20]. Figure 3 shows the lymphatic drainage pathways and the regional lymph nodes of the female genital organs [20].

### 2.5. Embryologic Assessment of the Lymphatic Drainage in the Median Compartment

#### Embryonic Origins of Lymphatic Vessels

**Figure 4:**
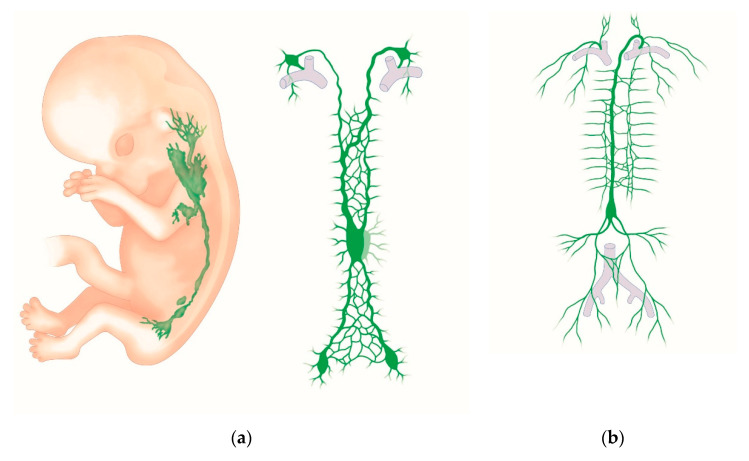
(**a**) Embryological development of the lymphatic system. Extreme left: schematic lateral view of an embryo in the eighth week of gestation, with three primitive lymph sacs. Middle: the lymphatic vessel system from the ventral aspect in a nine-week-old fetus, showing the pairwise thoracic duct. (**b**) Schematic illustration of the adult lymphatic system. The final thoracic duct and the right-sided lymphatic duct are also seen from the ventral aspect.

Malignancies of the uterus metastasize, as described earlier, by the lymphatic or hematogenic route, or by the direct invasion of neighboring structures. Since metastasis is mainly caused by lymphogenic tumor spread, and since the lymph drainage of the female genital tract (such as the uterus) is highly complex compared to other organs, the embryology of the lymphatic system will be considered in the following figure (Figure 4a,b). Embryologically, the female genital tract, the uterus, the fallopian tubes, and the upper part of the vagina arise from the paramesonephric ducts (Müllerian ducts). The distal fusion of the two ducts induces the development of the uterovaginal canal, the formation of the mesometrial tissue, and the broad ligaments. The fallopian tubes develop from the unfused cranial parts of the Müllerian ducts [19].

Historically, the investigation of the lymphatic vessels started in the 17th century. The anatomy of the large part of the lymphatic system had been described by the beginning of the 19th century. In 2010, Ribatti et al. presented an historical review of the embryonic origins of lymphatic vessels [21].

**Figure 5:**
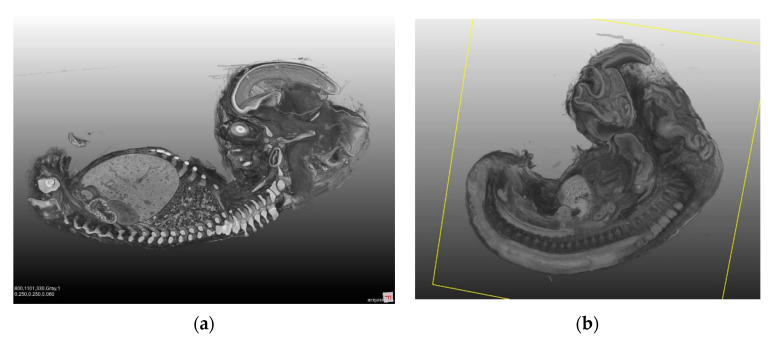
Lateral views of a human embryo obtained through computed tomography and magnetic resonance imaging. (a) Lateral view of an approximately-eight-week-old embryo. The human embro already has a visible human shape. However, the head is still disproportionately large, and accounts for nearly a half of the total length. The axial skeleton is formed. The protrusion of the abdomen is mainly caused by the large liver. The rudiments of the kidneys are seen in the caudal aspect. (**b**) A lateral view in an earlier stage of development. The embryo already has a characteristic C-shaped curvature.

The lymphatic system develops in close association with the venous system from the fifth week of gestation (Figure 4a) [22]. Endothelial cells grow from the cardinal veins into the surrounding mesenchyme. This occurs mainly at the origin of the internal iliac vein and the jugular vein. The newly-formed lymphatic vessels develop into bag-like structures, also known as lymph sacs. The left and right jugular lymph sacs, and the left and right posterior lymph sacs (iliac sacs) develop at this time. Between the sacs, a plexus of lymph vessels is formed along the dorsal thoracic wall. At the root of the mesentery, this plexus turns into an unpaired retroperitoneal sac. The chyle cistern is formed in front of the previously-mentioned retroperitoneal sac, in the region of the celiac trunk. The right lymphatic duct and the thoracic duct arise from the plexus of lymph vessels. The lymph system of the head, neck, and extremities develops from the posterior (iliac) lymphatic sacs and the jugular lymphatic sacs. Whether lymphatic vessels also arise directly from the mesenchyme due to vasculogenesis is a debated issue. Growth factor VEGF-C and the lymphoendothelial VEGF receptor 3 are essential for lymphangiogenesis. The lymph node colonization of mesenchymal bridges within the lymphatic sacs and larger lymphatic vessels gives rise to lymph nodes. Figure 4a,b provides a graphic overview of embryological development and the adult lymphatic system [22]. Figure 5a,b shows lateral views of a human embryo in various stages of development.

### 2.6. Anatomy-Based Methodology of Surgical Lymphadenectomy

Lymphatic drainage is achieved in endometrial carcinoma, as shown in the anatomical overview: on the one hand, through the pelvic lymph nodes, and on the other hand, through lymphatic pathways which can drain directly into the para-aortic regions through the adnexa. For this reason, para-aortic lymph nodes may be positive even in the presence of unaffected pelvic lymph nodes [19,20,22].

The pelvic lymph node groups include those of the obturator fossa, the lymph nodes in the region of the internal iliac artery and vein, the lateral and medial lymph nodes in the region of the external iliac artery and vein, and in the region of the common iliac artery and vein. In addition to the previously-mentioned vessels, the area of surgery encompasses other structures which must be visualized and protected. These include the ureter, the obturator nerve, the genitofemoral nerve with its femoral and genital branches, the lumbosacral trunk, and the superior hypogastric plexus in the region of the presacral lymph nodes [20,22]. In the obturator fossa runs the neurovascular obturator pedicle, which consists of the obturator nerve and the obturator vein and artery, which join the nerve coming from below. According to the location related to the obturator pedicle, lymph nodes in this area may be subdivided into supraobturator and infraobturator nodes [19].

The para-aortic lymph node groups include the cranial portions of the common iliac artery, the region of the caudal vena cava, including the aorta and the inferior mesenteric artery, and further cranially, the interaortocaval tissue extending to the renal pedicle, which becomes visible through the renal vein [20,22]. During para-aortic lymphadenectomy, the sympathetic trunk must be preserved also.

**Figure 6:**
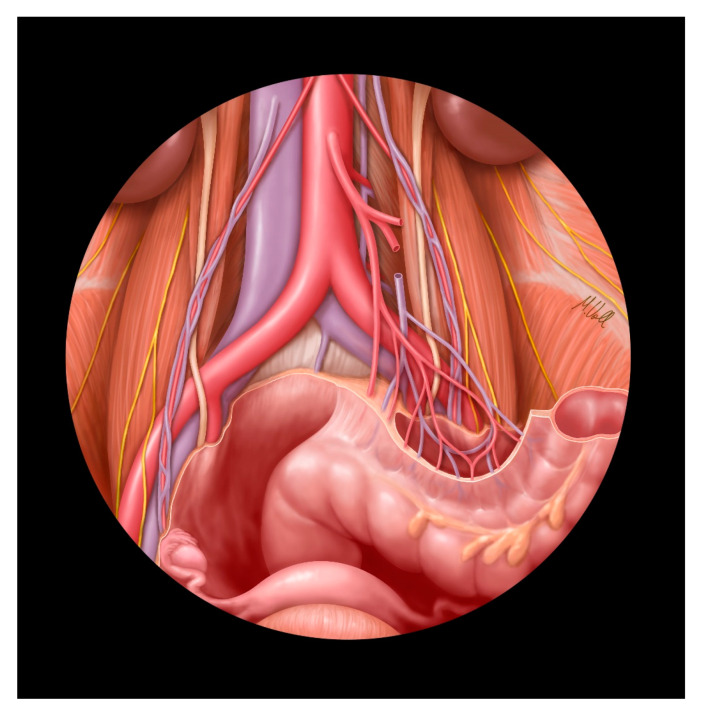
Schematic illustration of the anatomical area. This view is from the caudal–ventral aspect. The parietal peritoneum and the organs of the gastrointestinal tract to the sigmoid colon have been removed. The especially-complex anatomical area is on the left side, specifically the left common iliac artery and vein.

The challenge of cancer surgery is twofold: to strive for a maximum radical operation on the one hand, in order to ensure curative therapy, and to strive for the least loss of function on the other hand, in order to preserve the quality of life after surgery [19]. In the following, we highlight a specific anatomical area because of its topographical complexity (Figure 6). In front of the left side of the fourth lumbar vertebra, the common iliac arteries originate at the aortic bifurcation. They pass along the medial borders of the psoas major muscle, and divide into the internal and external iliac arteries. The common, internal, and external veins are located medial or dorsomedial to their arterial equivalents. The ureter is crossed posteriorly by the genitofemoral nerves, and anteriorly by the ovarian vessels. In addition, on the left side, the ureter crosses under the root of the sigmoid mesocolon and the inferior mesenteric pedicle. In the majority of cases, the ureter enters the pelvic cavity on the right side anterior to the external iliac artery, and on the left side anterior to the common artery [19]. Numerous sympathetic and parasympathetic autonomic nerves also contribute to the complexity of this region. These autonomic nerves must be preserved because they mediate anorectal and urogenital functions [19].

But why exactly this area? The lymph nodes that can be accessed with the least collateral damage are removed. Examples of these are the lymph nodes to the obturator nerve, but not further dorsally. Another example is the resection to the cross-over point of the renal vein, but not further cranially.

We only operate within accessible anatomical areas, and not in the corresponding compartments derived from anatomical or embryological studies.

### 2.7. Strategy of Lymphadenectomy

Lymphadenectomy has been referred to by various names in the published literature. As listed in Table 1, these include radical/complete or systematic lymphadenectomy, sentinel lymph node biopsy, therapeutic lymphadenectomy, lymph node debulking, and lymph node sampling.

The individual aspects are addressed here in detail.

## 3. Surgical Staging in Endometrial Cancer

The surgical management of endometrial cancer has undergone significant changes in the last few decades, including a change from open to minimally-invasive surgery. When the findings of the GOG#33 trial were published in 1998, the International Federation of Gynecology and Obstetrics (FIGO) introduced a shift from clinical to surgical staging, including lymphadenectomy [23,24]. Since this time, the question as to whether all patients need a lymphadenectomy, and the extent of the lymph node dissection have been the most controversial issues in gynecological oncology. In two randomized controlled trials published in 2008 and 2009, patients who received systematic pelvic lymphadenectomy were compared to those who did not undergo node dissection, and revealed no survival benefit in either group [14,15]. Both trials included patients with early-stage endometrial cancer. The data were evaluated critically because para-aortic lymph node dissection was not performed, and a rather small number of lymph nodes were excised [16]. A variety of surgical staging strategies were then used in gynecological oncology, ranging from complete bilateral pelvic +/− aortic lymph node dissection, random node sampling, and selected node dissection based on intraoperative frozen-section findings, to no lymph node dissection at all [17,18]. Sentinel lymph node mapping has gained increasing importance in recent times as an alternative concept to complete lymph node dissection.

### 3.1. Systematic or Complete Pelvic and Para-Aortic Lymph Node Dissection

Preoperative imaging studies to identify positive lymph nodes with techniques including magnetic resonance imaging, positron emission tomography or computed tomography have been unsatisfactory because of their poor sensitivity [25]. Surgical staging remains the gold standard for the assessment of lymph node involvement, and is performed by systematic pelvic and para-aortic lymph node dissection [26,27].

The revised FIGO staging system in 2009 divided stage IIIC endometrial cancer into IIIC1 (positive pelvic nodes) and IIIC2 (positive para-aortic nodes with or without positive pelvic nodes), reflecting the fact that the prognosis is worse when para-aortic lymph nodes are involved [24].

We still lack a standardized definition of adequate complete lymphadenectomy [12]. The current approaches include pelvic lymphadenectomy, para-aortic lymphadenectomy to the inferior mesenteric artery, and para-aortic lymphadenectomy to the renal vein [12].

The outcome of an international survey about the surgical management and adjuvant treatment of endometrial carcinoma throughout the world was published in 2015 [17]. Six-hundred and eighteen institutions around the world participated in this study. The indication for lymphadenectomy, anatomic limits, and extension were the main surgical issues. Of those centers at which lymphadenectomy was performed, 66% of the respondents conducted a systematic excision of lymph nodes, and 4% performed the sampling of the nodes. Both pelvic and para-aortic dissection were used (73.17%). Pelvic nodes alone were dissected by 15% of the respondents. The upper limit of the para-aortic dissection differed between the institutions: 7.9% of the respondents performed para-aortic dissection at the level of the inferior mesenteric artery, and 75.5% performed para-aortic dissection at the level of the renal vessels. In total, 2.9% of the respondents stated that they routinely resected lymph nodes in the suprarenal area. In Central Europe, lymphadenectomy was performed to the renal vessels in 86.8%, in USA/UK in 51.2%, in Asia in 80.8%, and in Southern Europe in 45.1% (*p* < 0.001) of cases [17].

The probability of lymph node metastasis across all FIGO stages is 15%.

A longer period of overall survival and fewer deaths were noted in patients with endometrial cancer who received pelvic lymphadenectomy combined with para-aortic lymph node dissection compared to those who received pelvic lymphadenectomy alone [28,29].

Three percent of the patients had isolated positive para-aortic lymph nodes and no positive pelvic lymph nodes. Of the 3%, 67–100% had lymph node metastases in a high para-aortic location, i.e., between the renal vein and the inferior mesenteric artery [30].

In a study performed by Mariani et al., 281 patients with endometrial cancer received lymphadenectomy. Twenty-two percent of patients with high-risk endometrial cancer had positive lymph nodes. Of the 22%, 51% had positive pelvic and para-aortic nodes, 33% only had positive pelvic lymph nodes, and 16% had isolated para-aortic nodes. Seventy-seven percent of patients with para-aortic nodes had metastases above the inferior mesenteric artery. These findings indicate that para-aortic lymphadenectomy up to the renal vein is advisable [31].

In terms of surgical boundaries for pelvic lymph node dissection, the published literature recommends the removal of lymphatic tissue from the deep circumflex iliac vein to the midpoint of the common iliac artery [30]. Complete para-aortic lymphadenectomy includes the removal of all nodal tissues and fat surrounding the aorta, inferior vena cava, and renal vessels, from the midpoint of the common iliac vessels caudally to the left renal vein cranially [32]. The left renal vein runs between the aorta and the origin of the superior mesenteric artery. The superior mesenteric artery needs to be preserved, because this artery provides the blood flow to the proximal of the transverse colon, the ascending colon, the cecum, the jejunum, the ileum, and the third portion of the duodenum [19].

In a multitude of solid malignancies, the lymph node count has become a marker of the adequacy of lymph node dissection. The results of two retrospective studies showed that patients with endometrial cancer had improved survival when 10–12 lymph nodes were removed [33,34]. The sampling of lymph nodes has a low sensitivity in endometrial cancer [12].

Systematic pelvic and para-aortic lymph node dissection enables the clinician to provide tailored adjuvant therapy and reduce adjuvant therapy-related morbidity [30].

According to the ESMO–ESGO–ESTRO guidelines, lymphadenectomy is not recommended in patients with low-risk endometrioid cancer. A systematic lymphadenectomy should be recommended to patients with major risk factors (grade 3 with deep myometrial invasion >50%) because of the higher prevalence of nodal metastasis in this population. In cases of intermediate-risk patients (deep myometrial invasion >50% or grade 3 superficial myometrial invasion >50%), the data have shown no survival benefit; a lymphadenectomy may be considered in this group for staging purposes [12].

### 3.2. What Is the Role of Sentinel Lymph Nodes in Endometrial Cancer?

Complete pelvic and para-aortic lymph node dissection is associated with major comorbidities, including lymphedema, lymphocysts, cellulitis, and damage to adjacent nerves. Furthermore, complete lymph node dissection is technically difficult in obese women, and the latter constitute a large part of patients with endometrial cancer [35].

Historically, the first successful instance of SLN mapping was reported in 1977; the procedure was a lymphangiography of the penis [36]. Since then, SLN mapping techniques have been investigated and developed for several other solid malignancies, including breast cancer and melanoma [37,38]. In gynecology, SLN mapping was first performed and accepted for patients with vulvar cancer. It is also promising in patients with endometrial and cervical cancer [35,39,40]. Although the concepts are similar, the approaches towards the standardization of the procedure differ because of differences in the incidence of the respective cancer, rates of lymph node metastasis, and the treatment or prognostic impact of the lymph node status for each disease [36].

As shown in Table 2, a number of tracers (indocyanine green, technetium, and blue) and various injection sites (cervical, subserosal myometrial, hysteroscopic peri-tumoral) have been described after SLN mapping was introduced for endometrial cancer [16,40,41]. ICG injected into the cervix emerged as the most consistently effective detection technique for endometrial cancer because of its high success rates and reproducibility [40].

SLN mapping must possess a high sensitivity and negative predictive value in order to be an acceptable staging method [36]. The FIRES trial, published in 2017, was a prospective multicenter cohort study in which sentinel lymph node mapping was followed by complete pelvic +/− para-aortic lymphadenectomy. Sentinel lymph node mapping with complete pelvic lymphadenectomy was performed in 340 patients, and para-aortic lymphadenectomy was performed in 196 patients (58%). Forty-one patients (12%) had positive nodes. SLN mapping had a sensitivity of 97.2% for the detection of node-positive disease, and a negative predictive value of 99.6%. The authors confirmed the high accuracy of sentinel lymph node mapping with the aid of indocyanine green for the detection of metastases, and conclude that SLN mapping might safely replace lymphadenectomy in the staging of endometrial cancer [35].

Pelvic sentinel lymph nodes follow two consistent lymphatic pathways: the upper paracervical pathway drains the medial external and/or obturator lymph nodes, and the lower paracervical pathway drains the internal iliac and/or presacral nodes. Furthermore, a non-pelvic pathway courses along the infundibulopelvic ligament to the para-aortic lymph nodes. SLN mapping for endometrial cancer revealed metastases in areas not usually included in a standard lymph node dissection. Sentinel lymph nodes are usually seen as ‘colored nodes’ or ‘radioactive nodes’ without regard to lymphatic anatomy [40]. In a prospective trial, How et al. examined the anatomical location of sentinel lymph nodes after the intraoperative cervical injection of tracers: the external iliac and obturator areas were the most frequent locations for SLN detection. Interestingly, positive SLNs were seen in the pre-sacral and parametrial regions, and around the internal iliac vein [40]. Metastatic lymph nodes in atypical regions were also reported by Geppert et al. [42]. Compared to standard lymph node dissection, SLN mapping increases the detection of overall metastases [43].

An increasing body of evidence suggests the non-inferiority of sentinel lymph node mapping compared to systematic lymphadenectomy in endometrial cancer [44]. In 2014, the National Comprehensive Cancer Network (NCCN) guidelines accepted SLN mapping as an alternative to complete lymphadenectomy in selected cases of endometrial cancer. In 2018, the NCCN extended the application of sentinel lymph node mapping to high-grade carcinomas [41]. Last updated in 2015, the ESMO–ESGO–ESTRO guidelines recommend the use of sentinel lymph node mapping only in controlled trials [12].

Recent data suggest that, in endometrial cancer, sentinel lymph node mapping does not influence the oncologic outcome of disease. Based on six studies comprising a total of 3536 patients, the authors of this meta-analysis concluded that, in terms of recurrence rates (any site and nodal recurrence) and the detection of positive para-aortic lymph nodes, sentinel node mapping is not inferior to standard lymphadenectomy. The overall recurrence rates revealed no significant difference (4.3% after sentinel node mapping and 7.3% after lymphadenectomy; *p* = 0.63). With regard to the detection of positive pelvic lymph nodes, the authors concluded that sentinel node biopsy may be considered superior to lymphadenectomy [44].

Sentinel nodes are processed according to an ultrastaging protocol. Pathologic ultrastaging (including immunohistochemical (ICH) staining and deeper serial sections) enhances the detection of malignant cells. The clinical significance of the increased detection of isolated tumor cells and micrometastasis is currently uncertain. Furthermore, the strategies used for the pathological investigation of SLNs vary among institutions and within the published data [36].

Future trials will have to address these questions and determine oncologic outcomes after the use of sentinel node mapping for endometrial cancer.

### 3.3. Therapeutic Pelvic and Para-Aortic Lymphadenectomy (tLNE)

Our extended knowledge of the embryological development of organ compartments, tissue boundary control, and their association with tumor spread and tumor progression has resulted in a new approach in cancer surgery: compartmental surgery in cancer, as established by M. Höckel in gynecological oncology [45].

According to this concept, tumor spread is initially restricted to permissive ontogenetic compartments and their corresponding lymph node basins. The complete surgical removal of these embryologically-defined areas by whole compartment resection with intact margins following ontogenetic planes will result in optimal tumor control [19]. Embryologically, the female genital tract, the uterus, the fallopian tubes, and the upper part of the vagina arise from the paramesonephric ducts (Müllerian ducts). The distal fusion of the two ducts induces the development of the uterovaginal canal, the mesometrial tissue, and the broad ligaments. The fallopian tubes develop from the unfused cranial portions of the Müllerian ducts. The lymphatic network of the Müllerian system derives from embryonal veins [19,46].

The transfer of this concept to endometrial cancer led to peritoneal mesometrial resection (PMMR) combined with pelvic and para-aortic therapeutic lymphadenectomy (tLNE) [45]. The lymphatic system plays a major role in tumor spread and progression. The first part of this review summarized the anatomy and embryonic origins of lymphatic vessels. Via lymph drainage, malignant cells are able to reach the blood circulation through the jugular veins, and cause hematogenic metastases. These mechanisms might be highly relevant in endometrial cancer, because the metastatic spread in this malignancy occurs predominantly through the lymphatic system [45]. There is convincing evidence that compartment resection, including the regional lymph compartment, reduces loco-regional recurrence even without adjuvant radiation [45,47,48,49,50].

Following TMMR and therapeutic lymphadenectomy without adjuvant radiation, Höckel et al. conducted a prospective single-center study comprising 305 cervical cancer patients with FIGO stage Ib to IIB disease, of whom 71 had positive lymph nodes; the authors noted recurrence-free and overall 5-year survival rates of 94% and 96%, respectively [51]. The preliminary evidence indicates that this concept yields comparable results even when it is used for endometrial cancer [52]. In cases of endometrial cancer, the compartmental concept could be equated with the Müllerian compartment, including the lymph compartments that drain regionally, and the so-called intercalating lymph nodes [52]. Kimmig et al. showed that PMMR with tLNE, performed by robotic-assisted laparoscopy, is a safe and feasible approach with low recurrence rates in patients with intermediate- and high-risk endometrial cancer [52]. The authors used indocyanine green (ICG)-enhanced fluorescence for the visualization of the Müllerian compartment and the subsequent lymphatic compartment. They injected ICG into the mid-corporal and fundal myometrium, and demonstrated two pathways for the transport of fluorescent lymphatic fluid. The first pathway was along the uterine vessels, passing the vascular mesometrium and reaching the pelvic lymph nodes along the internal and external vessels. The second was the ovarian mesonephric pathway to the para-aortic nodes. No drainage was observed along the ligamentous mesometrium (uterosacral ligament). The authors concluded that the number of patients (68) was too low to draw final conclusions about oncological outcomes, and that further multicenter trials will be needed in order to evaluate the value of compartmental surgery in endometrial cancer. A trial addressing this question is currently under way.

As described earlier, the lymphatic network of the uterus can be visualized by the injection of indocyanine green (ICG) as a guide in compartmental surgery. This is conceptually different from its use in sentinel lymph node detection. In a trial published in 2016, Kimmig et al. [45] investigated the intraoperative visualization of embryologically-defined organ compartments and their drainage after the injection of indocyanine green (ICG). Thirty-six patients with uterine cancer and no suspicious lymph nodes on macroscopic investigation participated in the study: 20 cervical cancer patients with FIGO stages Ib to IIB, and 16 endometrial cancer patients with FIGO stage I-III. Patients with endometrial cancer received PMMR (peritoneal mesometrial resection) with or without pelvic/para-aortic lymphadenectomy, and those with cervical cancer received TMMR (total mesometrial resection) and therapeutic pelvic lymphadenectomy. Prior to surgery, ICG was injected into the body of the uterus or the cervix. The authors showed that the lymphatic drainage differs according to whether ICG is injected into the cervix, the mid-corpus, or the fundus. The cervix drained along the caudal portion of the vascular mesometria, and along the ligaments. Fundal and mid-corporal drainage occurred by the mesonephric pathway along the ovarian vessels and the upper part of vascular mesometria [45]. The authors concluded that the visualization of the compartment and the lymphatic drainage system may assist the surgeon’s orientation intraoperatively, and may enhance the surgeon’s comprehension of the compartmental arrangement of the Müllerian system, as well as its borders to adjacent compartments. This may help to adapt surgery to individual circumstances. Furthermore, morbidity in the adjacent compartments of the bowel, bladder, ureter and nerves may be reduced [45].

## 4. Practical Aspects of Lymph Node Surgery

### Technical Challenges in Endoscopic Surgery

Several trials over the last few years have shown that minimally-invasive surgery is equivalent to open surgery with regard to the adequacy of the surgical resection and lymph node counts [53]. The additional positive effects of the minimally-invasive approach include a lower risk of intraoperative and postoperative complications (fewer wound-related complications, faster recovery, and earlier return to the activities of daily living) [53,54,55].

Within the spectrum of surgical laparoscopy, pelvic and paraaortic lymphadenectomy remain difficult high-end procedures. As described earlier, lymph node surgery in endometrial cancer is technically challenging, and can be time consuming because of the topographic complexity of lymphatic drainage as such, and the fact that the lymph nodes are directly adjacent to both blood vessels and nerves. Therefore, profound and exact knowledge of the anatomy is essential.

**Figure 7:**
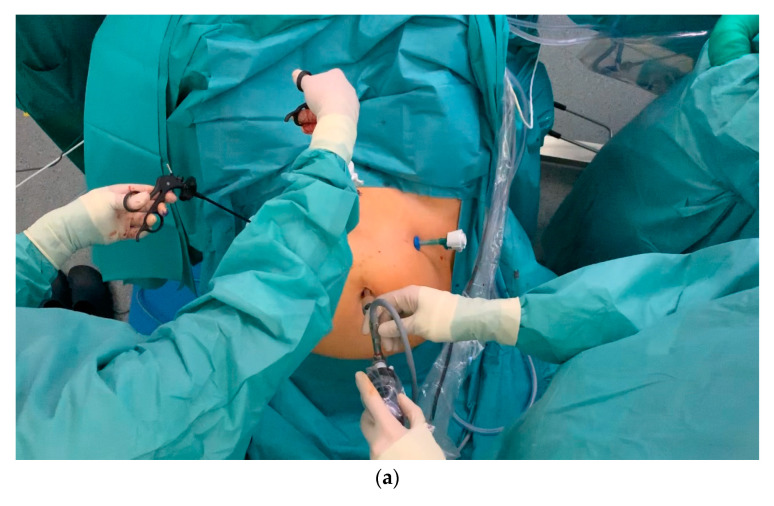

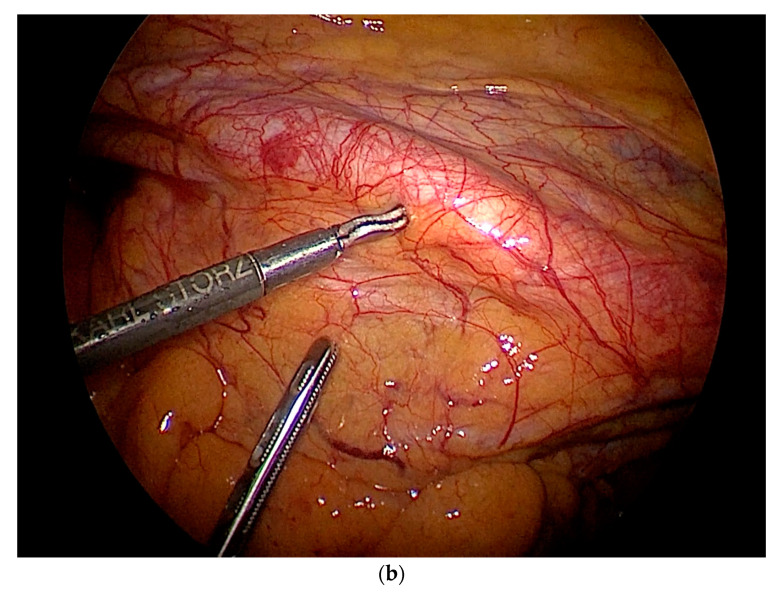
Trocar placement, the positioning of the surgical team, and operative findings. (**a**) The surgeon is to the patient’s left, and the first assistant to the patient’s right. The central trocar is inserted through the umbilicus. The working trocars are placed in the pelvis laterally and in the suprasymphysiary position. The first assistant holds the camera and uses the central trocar. The first assistant uses the right lateral trocar to provide assistance. The surgeon uses the left lateral and middle trocar. The middle working trocar should not be placed in the usual suprasymphysiary position, as this will inevitably cause the surgeon to work in his own direction. The middle trocar should be placed midway between the symphysis and the umbilicus. In order to avoid intraabdominal interference, the middle trocar should not be placed too close to the optical trocar. (**b**) Laparoscopic cranial view. Iliac vessels at the entrance to the right-sided pelvis. The ureter crosses the artery.

Figure 7 provides a detailed overview of the demanding requirements of endoscopic surgery, as well as the anatomical complexity of this area. As patients with endometrial cancer are often morbidly obese, performing a pelvic and paraaortic lymphadenectomy can be very challenging.

The following questions are yet to be answered conclusively:

- What is the most appropriate time to switch from the umbilical optical trocar to the suprasymphysiary trocar (perspective from above/below versus below/above)?
- What is the most suitable position for the working trocars with reference to the steps of surgery (perspective from above/below as well as below/above) so that the mutual angle of the instruments, as well as the mechanical actions of the surgeon, can be achieved smoothly and conventionally (i.e. not towards the surgeon but away from the surgeon)?

Conclusion: the course of the operation should not be oriented to the technical access and its limitations. Rather, it should be oriented towards the underlying anatomy and the embryological–oncological aspects.

## 5. Conclusions

Minimally-invasive surgery (conventional laparoscopy and robotic surgery) has rapidly replaced the open approach in endometrial cancer staging. Within the spectrum of surgical laparoscopy, pelvic and paraaortic lymphadenectomy remain difficult high-end procedures. Profound and exact knowledge of anatomy, including uterine lymphatic drainage, is essential for every surgeon. Lymph node assessment has also undergone significant changes after the introduction of SLN mapping. As in other diseases, the concept of ‘less is more’ has pervaded the treatment of endometrial cancer.

However, a number of significant questions remain unanswered, regarding the following: oncologic outcomes when sentinel lymph node mapping is used, the clinical management and impact of low-volume metastases in SLNs, and a consensus concerning the technique. These issues will have to be investigated in further large prospective trials.

## Figures and Tables

**Table 1:** Terms associated with lymphadenectomy.

Radical/Complete or Systematic Lymphadenectomy	Excision of All Lymph Nodes with Surrounding Fatty Tissue along the Vascular Pathways Corresponding to Lymphatic Flow in the Targeted Anatomical Region
Sentinel lymph node biopsy	Excision of preoperatively marked sentinel lymph nodes as the primary filtering point of lymphatic flow to the organ and the tumor
Therapeutic lymphadenectomy	Radical lymphadenectomy within the limits of embryonic anatomical development (as established by Michael Höckel)
Lymph node debulking	Reduction of tumor burden by the excision of enlarged lymph nodes in an advanced stage of cancer
Lymph node sampling	Unsystematic excision of separate, clinically unusual lymph nodes

**Table 2:** Characteristics of tracers for sentinel mapping in endometrial cancer [16,41].

Tracer Characteristics	ICG	Blue Dyes	Tc-99m
Injection	intraoperative	intraoperative	preoperative, including lymphoscintigraphy/SPECT
Signal duration	persistent	30 min	24 h
Costs	Low	Low	high
Allergic reactions	0.05%	2%	1–6/100,000
Other toxicity	None	color change of skin and urine, skin necrosis	radioactivity
